# A case report of post-traumatic eczema of the breast mimicking breast cancer: A diagnostic challenge

**DOI:** 10.1097/MD.0000000000048158

**Published:** 2026-03-20

**Authors:** Mingyue Yao, Zhaoyue Li, Anyang Liu

**Affiliations:** a Department of General Surgery, Beijing Tsinghua Changgung Hospital, School of Clinical Medicine, Tsinghua Medicine, Tsinghua University, Beijing, China.

**Keywords:** breast cancer, clinical mimicry, irritant contact dermatitis, mammary Paget disease (MPD), post-traumatic eczema

## Abstract

**Rationale:**

This case report describes a rare and severe presentation of post-traumatic eczema that clinically mimics mammary Paget disease. The case is uniquely significant due to its striking, “bark-like” hyperkeratotic appearance. This severity resulted from a combination of diagnostic delay and iatrogenic factors, specifically, prolonged, unsupervised postoperative application of povidone-iodine in a vulnerable elderly patient. This highlights the diagnostic challenge of differentiating between benign and malignant eczematous breast lesions.

**Patient concerns:**

An 81-year-old female with limited mobility developed a severe, crusted, lichenified eczematous lesion in her left breast following a surgical procedure. The condition persisted for 6 months without a correct diagnosis, resulting in a markedly severe presentation.

**Diagnoses:**

The initial clinical differential diagnosis was mammary Paget disease. However, histopathological examination confirmed a definitive diagnosis of post-traumatic eczema with a clear iatrogenic link to prolonged contact with povidone-iodine.

**Interventions:**

The patient was treated with topical corticosteroids and an oral antihistamine.

**Outcomes:**

The patient responded well to treatment, with complete resolution of the skin lesion. At the 1-year follow-up, there were no signs of recurrence.

**Lessons:**

This case underscores the critical importance of histopathology in differentiating inflammatory dermatoses of the breast from malignancies, as the clinical appearance can be highly misleading. Furthermore, it serves as a crucial cautionary example of the need for diligent and appropriate postoperative wound care management, particularly in vulnerable patient populations, to prevent severe iatrogenic complications.

## 1. Introduction

Post-traumatic eczema,^[1,2]^ also known as post-traumatic dermatitis, is an inflammatory skin reaction that occurs at the site of a previous injury, such as a laceration, burn, or surgical incision. Although the exact pathogenesis is not fully understood, it is thought to be a form of contact dermatitis or a localized hypersensitivity reaction triggered by trauma and subsequent wound care agents. When such lesions appear on the breast, they present a significant diagnostic dilemma, as they can mimic mammary Paget disease (MPD),^[3]^ a rare form of intraepidermal adenocarcinoma.

The diagnostic challenge is amplified by the stark contrast in prevalence. MPD is rare (1%–3% of all breast cancers),^[3]^ whereas eczema is common, affecting an estimated 2% of the adult population,^[4,5]^ but eczema occurring solely on the breast is uncommon. The significant clinical overlap—MPD often presents as an eczematous, scaly, pruritic eruption indistinguishable from dermatitis—frequently leads to misdiagnosis. This mimicry can result in diagnostic delays of 6 months or more, during which patients may receive inappropriate and ineffective topical treatments while the underlying malignancy progresses.^[6]^

Consequently, histopathology is not merely a confirmatory step but an essential and urgent diagnostic tool in this clinical scenario. The stakes of misdiagnosis are high and carry opposing risks. Misdiagnosis of a benign condition when MPD is present can delay life-saving cancer therapy, potentially allowing an underlying ductal carcinoma in situ or invasive ductal carcinoma to advance, thereby worsening the patient’s prognosis.^[6]^ Conversely, misdiagnosis of malignancy in a patient with benign dermatosis can lead to profound overtreatment, including unnecessary and irreversible procedures such as mastectomy. Such interventions can cause significant physical disfigurement, functional impairment, and substantial psychological harm. Therefore, a high index of suspicion and low threshold for biopsy are paramount for persistent, unilateral, eczematous breast lesions.

We present a case of severe, lichenified post-traumatic eczema of the breast that emphasized the diagnostic process and management.

## 2. Case presentation

Ethical Considerations This case report was conducted in accordance with the Declaration of Helsinki as revised in 2013. The Beijing Tsinghua Changgung Hospital Ethics Committee reviewed this case and determined that it was exempt from formal ethical approval because it was a retrospective single case report. Written informed consent was obtained from the patient and her family for the medical procedure and for the publication of this case report and any accompanying images. They are very pleased to share the disease progression and treatment course, hoping this report will raise clinical awareness and help prevent similar iatrogenic complications and diagnostic delays in other patients.

**Figure 1.**
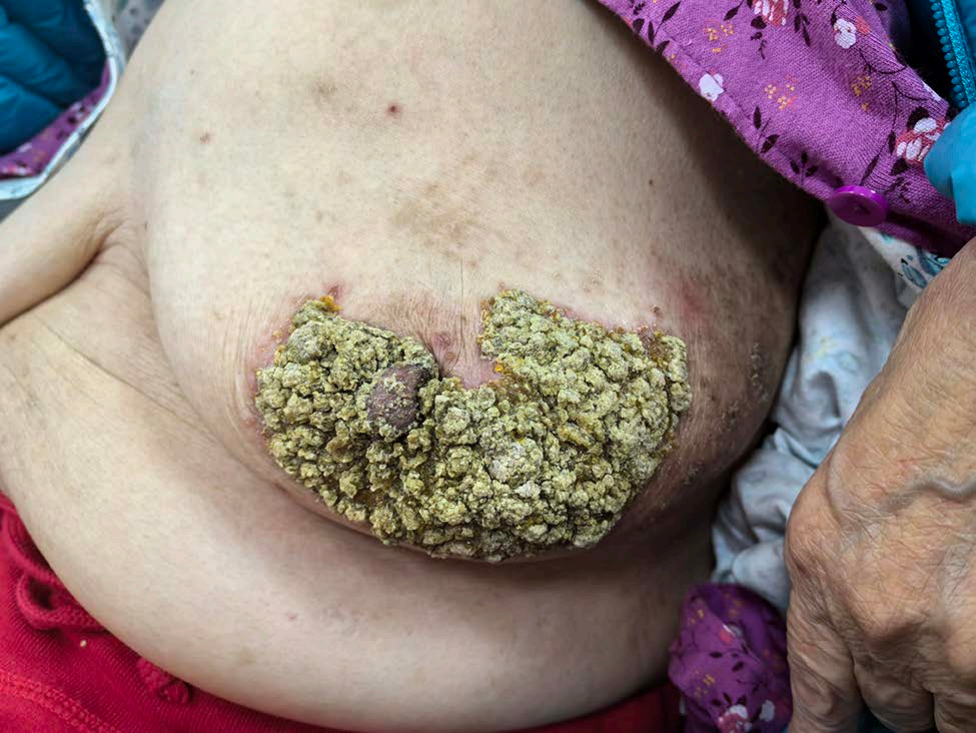
The appearance of the rash before treatment, a large, thickened plaque measuring approximately 8 × 10 cm was observed around the left areola.

An 81-year-old female presented to our outpatient clinic with a persistent rash on the left breast. Her medical history was significant for an incision and drainage procedure for a left para-areolar abscess performed at our hospital 6 months prior to presentation. Owing to the patient’s limited mobility, her family managed postoperative wound care at home, which involved prolonged application of povidone-iodine for disinfection. During the recovery period, the patient developed a mild pruritic rash at the surgical site. She reported mild pruritus (itching) but denied significant pain or bleeding and did not seek immediate medical attention. Over the following months, the rash progressively worsened, expanding in area and forming thick dry crusts, giving it a “bark-like” appearance (Fig. 1). A detailed timeline of the patient’s clinical course is presented in Table 1.

**Table 1:** Timeline of patient’s clinical course.

Time point	Event
−6 mo	Patient undergoes incision and drainage for a left para-areolar abscess.
−6 to −1 mo	Postoperative period. Family manages wound care with prolonged daily application of povidone-iodine at home due to patient’s limited mobility.
~ −5 mo	Onset of a mild, pruritic rash around the surgical site.
−5 mo to day 0	The rash progressively worsens, expanding in area and forming thick, dry, hyperkeratotic crusts.
Day 0	Patient presents to the outpatient clinic for evaluation of the persistent breast lesion.
Day 1	The thick crusts are debrided using an occlusive dressing of erythromycin ointment. Two **full-thickness excision** biopsies are performed for histopathological analysis and fungal culture.
Day 7	Histopathology report confirms benign inflammatory changes with no evidence of malignant cells. Fungal culture is negative. Diagnosis of post-traumatic eczema is established.
Day 7	Treatment is initiated with topical fluticasone propionate cream and oral ebastine (10 mg, once daily).
Day 14 (1-wk follow-up)	The lesion shows significant clinical improvement, with complete resolution of exudation and marked reduction in scaling and erythema.
+1 yr (1-yr follow-up)	The patient remains asymptomatic with no signs of recurrence of the lesion.

On clinical examination, a large, thickened plaque measuring approximately 8 × 10 cm was observed around the left areola. The lesion was distributed in the periareolar region, and extended inferiorly and bilaterally. The plaque was characterized by severe hyperkeratosis and lichenification. The texture was notably rough and firm, with thick, dry, adherent yellowish-brown crusts. The lesion was highly suspicious for eczematous-like breast cancer (Paget disease) given its location and chronic nature. A review of the systems was negative for constitutional symptoms such as fever, chills, night sweats, or unintentional weight loss, and the patient reported no other skin lesions or systemic complaints, which argued against a systemic inflammatory or malignant process. Severe crusting was attributed to the long delay in seeking treatment, allowing for cycles of exudation, drying, and scaling.

**Figure 2.**
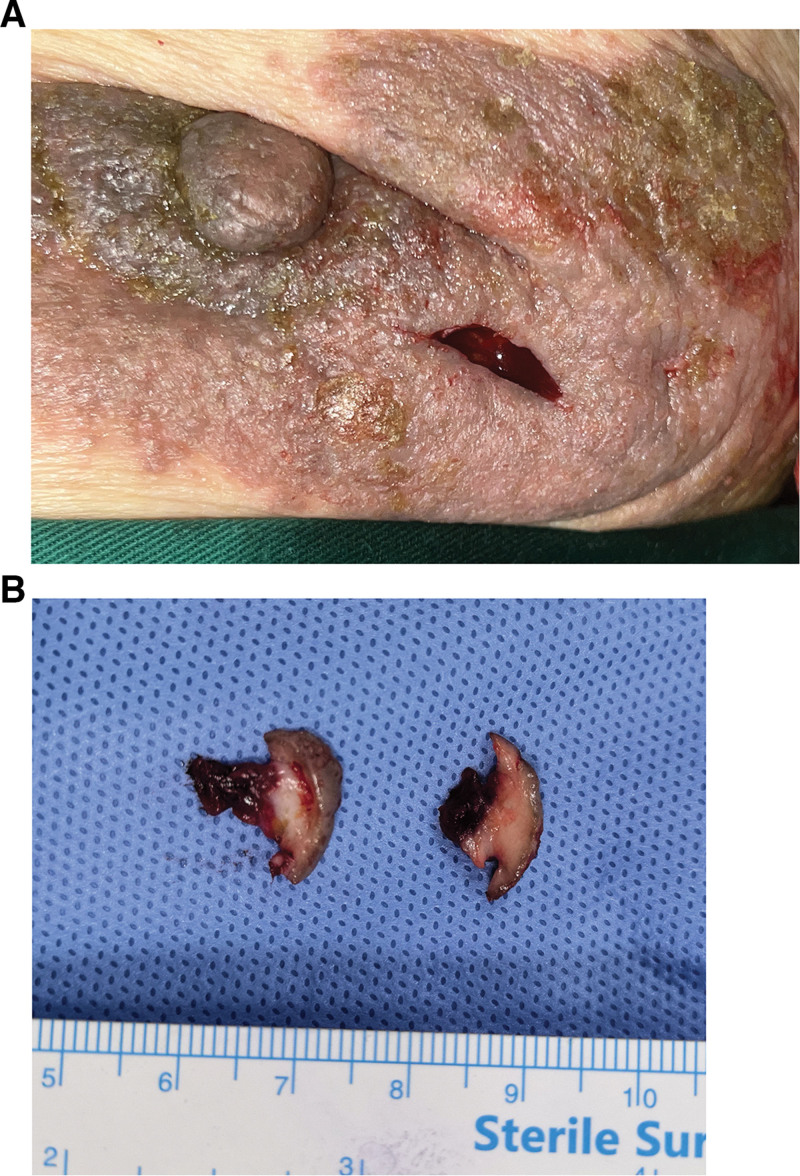
(A) The appearance after thick crust removal shows red rashes. (B): Two specimens obtained through full-thickness skin and subcutaneous tissue resection for biopsy.

To proceed with the diagnosis, the thick crusts were first removed by applying an occlusive dressing of erythromycin ointment, which revealed red and uneven rashes (Fig. 2A). Following debridement, 2 full-thickness skin and subcutaneous tissue excision biopsies were performed under local anesthesia. The specimens, measuring approximately 1 × 1 cm and appearing grossly normal (Fig. 2B), were sent for histopathological examination and fungal cultures.

The pathology report revealed acanthosis, spongiosis, and perivascular lymphohistiocytic infiltration in the dermis, consistent with eczematous dermatitis. Crucially, no malignant cells were identified in hematoxylin and eosin (H&E)-stained sections. Immunohistochemical (IHC) staining was performed to exclude MPD. The rationale for selecting cytokeratin 7 (CK7) and epithelial membrane antigen (EMA) is their high sensitivity for identifying the glandular differentiation characteristic of Paget cells, distinguishing them from reactive keratinocytes. The results showed that the epidermal cells were negative for CK7 and EMA markers, typically associated with Paget cells, further confirming the benign nature of the lesion. Given the conclusive negative results for these primary adenocarcinoma markers and the inflammatory morphology on H&E, additional stains (e.g., HER2) were deemed unnecessary. Furthermore, the fungal culture was negative.

**Figure 3.**
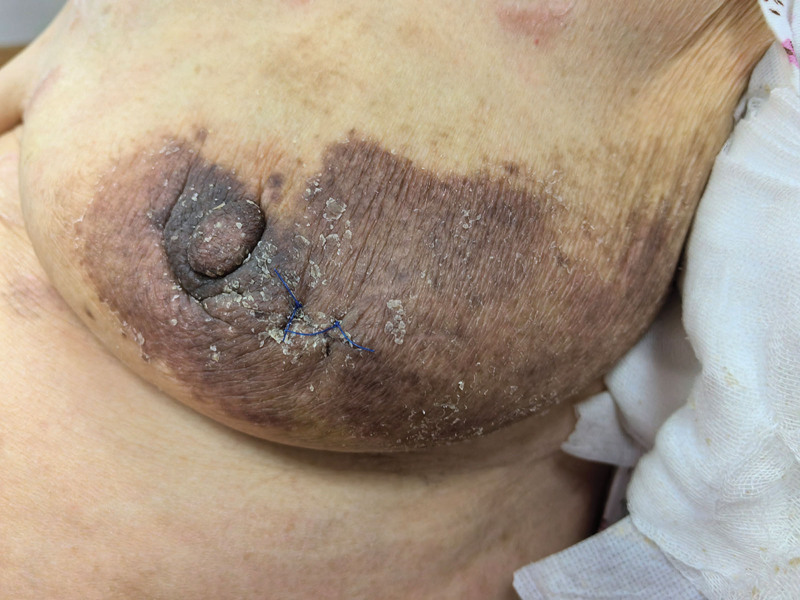
The appearance after 1 wk of treatment, already dry with no exudation.

Based on the clinical history and exclusion of malignancy and fungal infection, a final diagnosis of post-traumatic eczema, likely a severe form of irritant contact dermatitis (ICD), was made. The patient was treated with topical fluticasone propionate cream applied to the affected area and an oral ebastine (10 mg once daily). After 1 week of treatment, the rash showed significant improvement, with resolution of exudation and scaling (Fig. 3). The patient was followed up for 1 year with no signs of recurrence.

## 3. Discussion

This case illustrates a challenging diagnostic scenario in which a benign inflammatory condition clinically mimics cutaneous malignancy. The patient’s delayed presentation led to a more severe crusted lesion, which complicated the initial assessment. This underscores the importance of timely evaluation of persistent skin changes in the breast, especially after surgery or trauma.

### 3.1. Differential diagnosis

The primary diagnostic challenge was to differentiate this severe eczematous lesion from several other conditions, most importantly, MPD.

**MPD:** This was the primary differential diagnosis, as the lesion involved the nipple-areola complex and was unilateral, chronic, and eczematous in nature, closely resembling MPD.^[7]^ Furthermore, approximately 50% of patients with Paget disease present with a palpable underlying breast mass,^[3]^ which was absent in the present case. A definitive exclusion was made by biopsy, as histopathology showed no evidence of intraepidermal Paget cells, and immunohistochemistry (IHC) was negative for both CK7 and EMA. While H&E morphology is often suggestive, immunohistochemistry is the gold standard for differentiating these 2 conditions. Paget cells of glandular origin were characteristically positive for CK7 and EMA, whereas keratinocytes were negative. In our case, negative staining for both CK7 and EMA was pivotal in confidently excluding Paget disease and securing the correct diagnosis of eczema.

**Common Inflammatory Dermatoses:** While final diagnosis was a form of eczema, typical atopic eczema is often bilateral and associated with a personal or family history of atopy. It was excluded here based on the unilateral distribution and lack of atopic history.^[8]^ Psoriasis typically manifests as well-defined, erythematous plaques with a characteristic silvery scale, which is absent here and rarely isolated to the breast without involvement of extensor surfaces such as the elbows and knees.^[9]^ Thus, it was excluded due to the atypical morphology and the absence of involvement at typical sites. A clinical history of onset after trauma and exposure to a chemical agent strongly indicated contact-type dermatitis.

**Superficial Fungal Infection:** Tinea corporis can present as an erythematous, scaling plaque but often demonstrates a raised, active border with central clearing.^[10]^ This diagnosis was definitively excluded from the negative fungal culture.

**Bowen Disease:** This condition, a form of squamous cell carcinoma in situ, can present as a slow-growing, well-demarcated, scaly erythematous plaque and can be clinically indistinguishable from eczema or MPD.^[11]^ This was excluded by histopathological examination (negative histopathology), which showed no evidence of keratinocyte atypia or dysplasia, further reinforcing that biopsy is nonnegotiable for such persistent lesions to rule out malignancy.

### 3.2. The role of povidone-iodine in irritant contact dermatitis

The development of eczema following skin trauma is a well-known phenomenon. The prolonged use of povidone-iodine^[12]^ by the patient’s family for wound dressing may have acted as a sensitizing agent. This exposure likely contributed to a delayed-type hypersensitivity reaction or, more likely in this case, severe ICD that manifested as eczema. Although povidone-iodine is a widely used antiseptic with a generally low irritant potential upon brief contact, its mechanism of action involves the slow release of free iodine. When the solution remains wet on the skin for an extended period, the continuous release of free iodine can cause significant chemical and oxidative damage. This leads to a severe inflammatory reaction that mimics a chemical burn. This phenomenon is well-documented in the postsurgical setting, where pooling of the antiseptic in dependent areas due to patient positioning or prolonged contact under occlusive drapes can provoke severe ICD.^[13–15]^ In our patient, limited mobility and daily application of povidone-iodine by caregivers created a high-risk environment that precipitated this reaction. The antiseptic likely pooled against her skin for prolonged periods, leading to the severe hyperkeratotic reaction. This case highlights the critical intersection of iatrogenic complications with the patient’s social and physical circumstances. It underscores that postoperative care instructions must be not only clear, but also practical and safe for a specific patient’s home environment and caregiver capabilities, especially for vulnerable individuals with limited mobility.

The successful and rapid response to topical steroids and antihistamines further supported the diagnosis of an inflammatory process rather than malignancy.

### 3.3. Clinical learning points

This case provides several critical learning points for clinicians:

#### 3.3.1. *Persistent breast dermatitis warrants biopsy*

Any unilateral eczematous lesion of the breast or nipple-areola complex that is persistent or unresponsive to standard topical therapy must be considered a potential malignancy until proven otherwise. A low threshold for performing skin biopsy is essential to ensure the timely diagnosis of MPD and prevent the potentially devastating consequences of delayed cancer treatment.

#### 3.3.2. *Overuse of antiseptics can provoke severe dermatitis*

Clinicians and caregivers must recognize that topical antiseptics, such as povidone-iodine, are potent chemical agents. Instructions for postoperative wound care should be explicit about the correct frequency and amount of application. They must also emphasize the importance of allowing the solution to dry completely to prevent pooling and prolonged wet contact, which can cause severe ICD.

#### 3.3.3. Early dermatology referral may avoid prolonged suffering

In cases of persistent or atypical dermatological presentations, particularly in an anatomically sensitive and high-stakes area such as the breast, early consultation with a dermatologist is essential. Such consultation can facilitate accurate diagnosis, guide appropriate management, and prevent the prolonged patient morbidity and diagnostic uncertainty exemplified in this case.

### 3.4. Limitations

This study has some limitations. As this was a single case study, the findings cannot be generalized. Furthermore, it cannot fully determine causality regarding the exact contribution of trauma versus the antiseptic agent. The clinical presentation was the result of a specific and unusual confluence of factors (postsurgical trauma, iatrogenic antiseptic overuse, and prolonged diagnostic delay), making this a rare variant of post-traumatic eczema. This case also highlights diagnostic challenges that are often amplified in resource-limited settings where access to specialized dermatopathology and immunohistochemistry may be delayed, emphasizing the need for high clinical vigilance.

## Acknowledgments

The authors thank the patient for providing consent for the publication of this case.

## Author contributions

**Conceptualization:** Anyang Liu.

**Data curation:** Mingyue Yao.

**Investigation:** Mingyue Yao.

**Project administration:** Zhaoyue Li.

**Software:** Zhaoyue Li.

**Supervision:** Anyang Liu.

**Writing – original draft:** Mingyue Yao, Anyang Liu, Zhaoyue Li.

**Writing – review & editing:** Anyang Liu, Zhaoyue Li.
